# Case of a 16‐Year‐Old Boy With Headache, Vomiting, and Behavioral Changes

**DOI:** 10.1002/acn3.70052

**Published:** 2025-05-07

**Authors:** Kundian Guo, Zhen Hong

**Affiliations:** ^1^ Department of Neurology West China Hospital, Sichuan University Chengdu Sichuan China; ^2^ Institute of Brain Science and Brain‐Inspired Technology of West China Hospital Sichuan University Chengdu Sichuan China; ^3^ Department of Neurology Chengdu Shangjin Nanfu Hospital Chengdu Sichuan China

interACTN Case #46: Available: https://interactn.org/2025/04/02/case‐46‐the‐case‐of‐a‐16‐year‐old‐boy‐with‐headaches‐vomiting‐and‐behavioral‐changes/

## 1 Summary of Case

**FIGURE 1:**
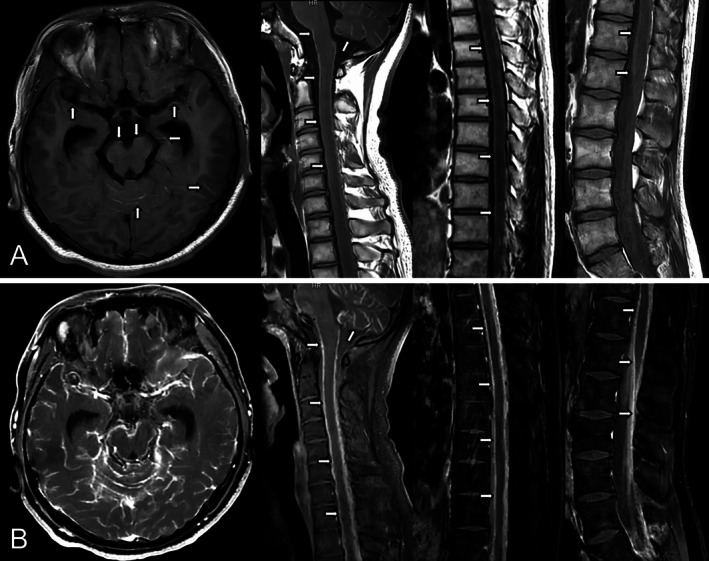
MRI brain and spine. (A) Non‐contrast T1‐weighted images show hyperintensities along sulcal spaces and the surface of the spinal cord (arrows). (B) Post‐contrast T1‐weighted images show diffuse irregular and continuous leptomeningeal enhancement, without any skip area from the cerebral hemispheres to the conus medullaris (arrows). Hydrocephalus is revealed.

**FIGURE 2:**
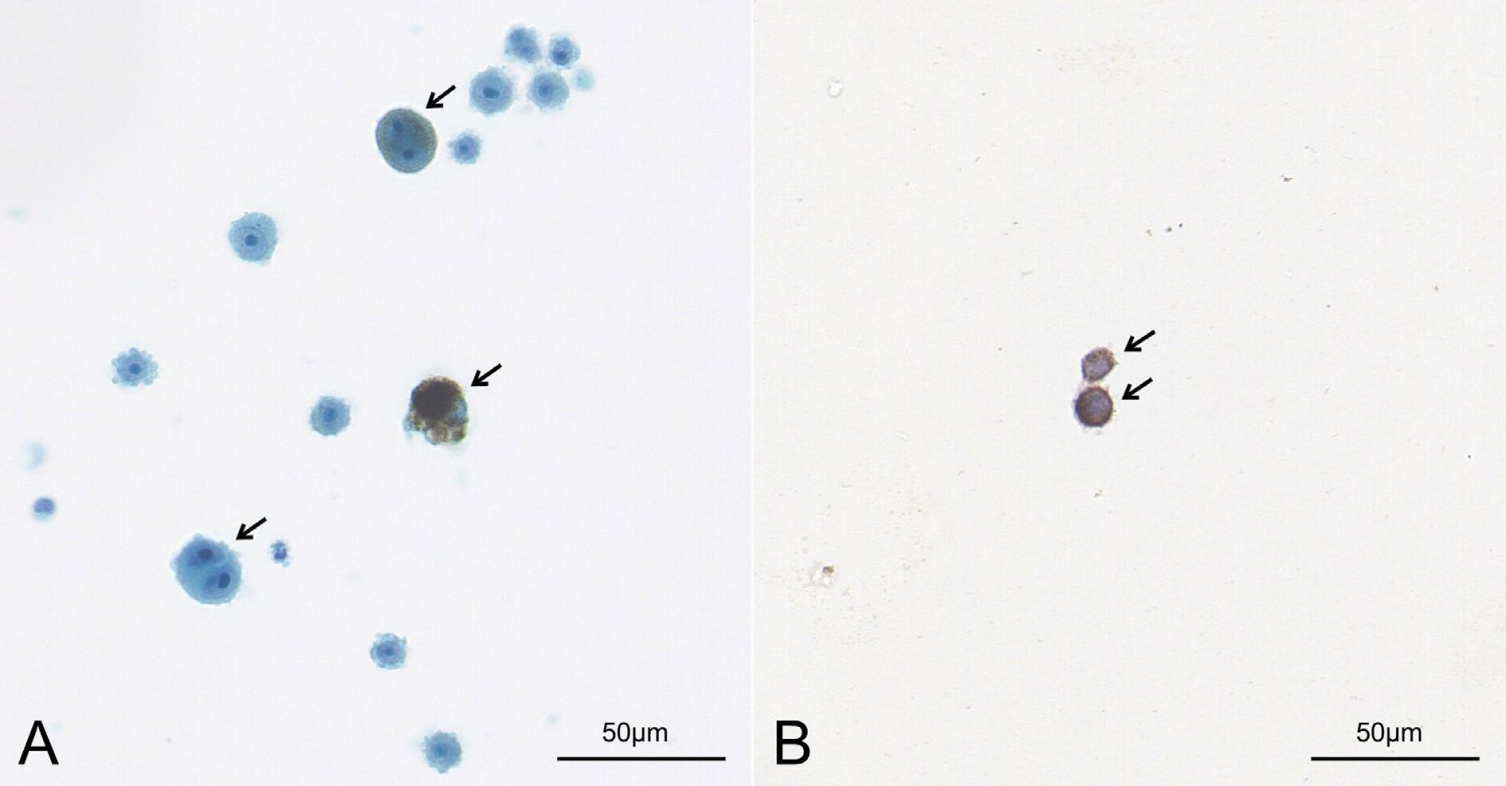
CSF cytopathology. (A) Papanicolaou‐stained CSF cytospin shows malignant cells with vesicular nuclei, prominent macronucleoli, and cytoplasmic melanin pigment. Mitotic figures are seen in some malignant cells (arrows, ×400). (B) The malignant cells are immunopositive for HMB‐45 (arrows, ×400).

A 16‐year‐old boy without any known medical history presented with headaches, vomiting, and behavioral changes. He was in his usual state of health 3 months before admission until the symptoms initially started as headaches and nausea. The headaches got worse when he lay down. The headaches kept worsening but were still manageable with NSAIDs until 1 month before admission. He began vomiting when the headaches were severe. He also felt stiff in his neck. His family noticed that he became irritable. They also noticed that he had irrelevant or incomprehensible talk sometimes. On neurological examination, the patient was alert and well‐oriented with intermittent behavioral changes in the form of agitation. His speech was clear but sometimes irrelevant. Reflexes were decreased (1+) but symmetric in the upper and lower extremities. Meningeal irritation signs were revealed. Brain and spine MRI showed hyperintensities along sulcal spaces and the surface of the spinal cord on unenhanced T1‐weighted images with diffuse leptomeningeal enhancement involving the entire neuraxis on post‐contrast enhanced images (Figure 1). Cerebrospinal fluid (CSF) analysis demonstrated mild pleocytosis (20 cells/mm^3^, all lymphocytes) with elevated protein (3.77 g/L). CSF cytopathology revealed malignant cells containing melanin pigment, staining positively for melanocyte‐specific markers, including HMB‐45, Mart‐1, and S‐100 on immunocytochemistry (Figure 2). Ocular and dermatological examination and whole‐body PET found no evidence of nevi or melanoma in other body parts.

## 2 Diagnosis

Primary diffuse leptomeningeal melanomatosis (PDLM).

## 3 Take‐Home Points

- PDLM is a rare primary melanocytic neoplasm of the CNS derived from melanocytes originating in the neural crest [1].
- Hyperintensity of the leptomeninges on unenhanced T1‐weighted images is an important imaging clue leading to the diagnosis of PDLM [2].
- Thorough examinations, including dermatological and ocular examination as well as whole‐body FDG‐PET, should be performed to exclude neurocutaneous melanosis and leptomeningeal metastases from a non‐CNS melanoma [1].
- Histopathology with meningeal biopsy is required for a definitive diagnosis of PDLM; however, the diagnosis can also be established with a positive CSF cytology [3].

## Author Contributions

Kundian Guo and Zhen Hong designed the case report, collected data, and consented the patient for participation. Kundian Guo drafted the manuscript. Zhen Hong revised the manuscript.

## Conflicts of Interest

The authors declare no conflicts of interest.

## Data Availability

The de‐identified data that support the findings of this study are available from the corresponding author upon reasonable request and in accordance with current legislation regarding the protection of personal data.
